# Game Theory-Based Signal Control Considering Both Pedestrians and Vehicles in Connected Environment

**DOI:** 10.3390/s23239438

**Published:** 2023-11-27

**Authors:** Anyou Wang, Ke Zhang, Meng Li, Junqi Shao, Shen Li

**Affiliations:** Department of Civil Engineering, Tsinghua University, Beijing 100084, China

**Keywords:** signal control, game theory, Nash bargaining, pedestrians, SUMO

## Abstract

Signal control, as an integral component of traffic management, plays a pivotal role in enhancing the efficiency of traffic and reducing environmental pollution. However, the majority of signal control research based on game theory primarily focuses on vehicular perspectives, often neglecting pedestrians, who are significant participants at intersections. This paper introduces a game theory-based signal control approach designed to minimize and equalize the queued vehicles and pedestrians across the different phases. The Nash bargaining solution is employed to determine the optimal green duration for each phase within a fixed cycle length. Several simulation tests were carried out by SUMO software to assess the effectiveness of this proposed approach. We select the actuated signal control approach as the baseline to demonstrate the superiority and stability of the proposed control strategy. The simulation results reveal that the proposed approach is able to reduce pedestrian and vehicle delay, vehicle queue length, fuel consumption, and CO_2_ emissions under different demand levels and demand patterns. Furthermore, the proposed approach consistently achieves more equalized queue length for each lane compared to the actuated control strategy, indicating a higher degree of fairness.

## 1. Introduction

### 1.1. Research Background

As urbanization continues to accelerate, traffic congestion is becoming more and more serious, particularly in large cities. According to an INRIX global traffic scorecard report [1], the estimated cost per driver in London due to congestion is reaching GBP 1377, and the hours lost in congestion are 156. Intersections, as one of the most crucial components of the transportation system, are where congestion is most likely to occur.

In recent years, with the development of Internet of Things (IoT) technology, various data acquisition has become more convenient and rapid. Vehicular ad hoc networks (VANETs), as a branch of the IoT, have facilitated direct communication between vehicles (V2V) and between vehicles and infrastructure (V2I) [2], which enables vehicles to promptly send and receive current traffic information. Therefore, VANETs can be employed to acquire real-time vehicle information and road conditions at intersections, such as the speed and position of vehicles [3,4]. Moreover, other devices, such as GPS, Bluetooth, loop detectors, and cameras installed at intersections, can also collect traffic data to compensate for the limitations of VANETs [5].

Intersections provide orderly movements for pedestrians and vehicles by means of signal control, and only a portion of non-conflicting pedestrians and vehicles are allowed to pass the intersection at the same time; thus, a suitable signal control strategy is critical to the efficiency and safety of intersection movements. Intersections in metropolitan areas and major urban arterials with high pedestrian demands are commonly encountered in daily life. Consequently, several studies have been conducted to determine the optimal control strategies for signalized intersections, taking into account both vehicles and pedestrians.

### 1.2. Signal Control Considering Pedestrians

Ishaque et al. [6] examined the effects of cycle length on both pedestrian and vehicle delays through a microsimulation model. Their study demonstrated that the optimal cycle length can be determined by considering the relative proportion of different traffic participants. Li et al. [7] developed a signal optimization strategy to minimize the weighted delay of pedestrians and vehicles. They used a Japanese intersection as a case study to evaluate the performance of the proposed model. The results indicated that the average delay per person can be improved by 10% without changing the existing cycle length. Similarly, Liang et al. [8] also formulated their objective as minimizing the weighted delay of pedestrians and vehicles. They utilized a genetic algorithm to optimize the signal phase sequences and validated their approach using simulation software. Further studies conducted by Yang et al. [9] and Ma et al. [10] primarily focused on contrasting the distinctions between an exclusive pedestrian phase and a conventional two-way crossing in terms of efficiency and safety. Yu et al. [11] proposed a convex programming approach to optimize signal timings considering both pedestrians and vehicles. The results of this study showed that a two-stage crosswalk may outperform a one-stage crosswalk in terms of both vehicle and pedestrian delays in some circumstances. Xu et al. [12] conducted multi-intersection traffic signal control research, taking into account both pedestrians and vehicles using reinforcement learning. Their study focused on efficiency, safety, and scalability. Yazdani et al. [5] also proposed a real-time signal control approach based on reinforcement learning, considering both pedestrian and vehicle flows. Their objective was to minimize total user delay in an isolated intersection. Additionally, they addressed pedestrian–vehicle interactions and the phenomenon of jaywalking.

### 1.3. GT-Based Signal Control Studies

Game theory (GT) is an innovative approach to solving traffic signal control problems, as it provides a flexible framework for modeling, optimizing, and managing complex traffic systems. GT has a wide range of applications in economics, military, and artificial intelligence. Its capacity to account for strategic interactions, uncertainties, and multiple objectives makes it a valuable tool in improving traffic flow, reducing congestion, and enhancing overall transportation efficiency. In the field of transportation, GT has been widely used in congestion pricing [13], route choice [14], and mode choice modeling [15]. To the best of our knowledge, Ling Long et al. [16] were the first to apply GT in traffic signal control. They modeled a two-phase intersection as a two-player cooperation model and then utilized the Nash bargaining (NB) solution to obtain the optimal control strategy. Similarly, Abdelghaffar et al. [17] also modeled a signalized four-phase intersection as a four-player cooperation game to obtain optimal control strategy by considering a variable phasing sequence and free cycle length. Additionally, they also extended their work to the network level [18]. Furthermore, Elouni et al. [19] compared the operation of game theoretic decentralized and centralized traffic signal controllers. Dong et al. [20] focused on multi-intersection. They applied a two-person static game model to the multi-intersection coordinate control problem. The Nash equilibrium concept was utilized to minimize vehicle delay, and simulation results demonstrated the superiority of the proposed model compared with a fixed-time signal control strategy. Another study [21] formulated the signal control problem using the Stackelberg approach with the objective of minimizing the queuing delay, ultimately solving it using the Nash equilibrium. Multiagent systems were also combined with GT to solve traffic signal control problems. Xia et al. [22] used GT to address coordination between agents based on traffic signal control with Q-learning. Daeichian et al. [23] employed fuzzy Q-learning and GT to form policy based on previous experiences and the decisions of neighbor agents under a classical non-stationary environment with the objective of reducing average vehicle delay. Abdoos [24] developed traffic signal controllers for multiple intersections using multiagent systems. Each intersection is controlled by an agent using Q-learning, and GT is employed to determine how to cooperate between agents. The simulation results indicated that the proposed method can effectively reduce the average vehicle delay. Babatunde et al. [25] recently developed a fuel-based signal controller from the perspective of the environment, with an objective function that combines operational measures and fuel consumption. In order to reduce fuel consumption at a signalized intersection, the optimal signal timing is obtained by the Nash bargaining solution.

Traffic demand is significantly influenced by various factors, such as time of day, day of the week, weather, accidents, etc., making the traffic demand at intersections highly stochastic in reality. In such an environment, a traditional fixed-time signal control strategy cannot adapt to the changing traffic demand, potentially leading to congestion at intersections. To explore the impact of stochastic traffic demand on signal control, several studies [26,27,28,29] have been conducted to determine the optimal signal control strategy under a stochastic dynamic traffic environment. From these studies, it can be concluded that in a stochastic traffic environment, the real-time adaptive signal control strategy can achieve good results.

From the literature research, it is evident that many scholars have delved into the realm of signal control using GT, predominantly concentrating on the “vehicles” perspective. The real-time adaptive signal control strategy has proven effective in addressing the challenges posed by stochastic traffic demand. In light of this, this study puts forth an innovative real-time adaptive signal control approach based on GT, tailored to a connected environment. Notably, our approach takes both vehicles and pedestrians into consideration at an isolated signalized intersection. We assume that the real-time speed and location information of vehicles can be obtained through VANETs, while pedestrians waiting at the intersection can be detected by cameras. We apply a four-player GT approach to determine the optimal green duration for each phase at the intersection through NB within a fixed cycle length. To assess the effectiveness of our proposed approach, we rigorously evaluate its performance through simulation tests conducted using SUMO software. The contributions of this paper to the literature are as follows:

1. We introduce an NB-based game-theoretic signal control approach, taking pedestrians into consideration for the first time (to the best of our knowledge), with the objective of minimizing and equalizing queued vehicles and pedestrians across the different phases;
2. Various demand levels and demand patterns have been tested to demonstrate the effectiveness, superiority, and stability of the proposed NB signal control approach in comparison to the actuated signal control;
3. We also take conflicts between pedestrians and right-turning vehicles into consideration, conducting a sensitivity analysis on right-turning vehicles to reveal the superiority of the proposed NB signal control approach.

The remainder of this paper is organized as follows. The next section introduces the materials and methods. The simulation tests and results are provided in Section 3. Finally, the conclusions, limitations, and future work are shown in Section 4.

## 2. Materials and Methods

### 2.1. Problem Definition

In a four-phase isolated intersection, signal control is typically necessary to coordinate the flow of vehicles and pedestrians to ensure traffic safety and efficiency. In this paper, we propose a novel real-time adaptive signal control approach in a connected environment, with the assumption that the speed and location information of vehicles can be obtained through VANETs, while pedestrians waiting at the intersection can be detected by cameras. Within the objective of minimizing and equalizing both vehicle and pedestrian queue length at an isolated signalized intersection, the proposed NB approach determines the optimal green duration for each phase. The cycle length remains fixed, the sequence of phases remains unchanged, and the green duration for each phase must fall within the minimum and maximum green duration limits.

### 2.2. Game Theory and Nash Bargaining

GT is dedicated to addressing real-world challenges characterized by elements of conflict and cooperation. The formulation of a game requires the inclusion of three fundamental components: players, strategies, and payoff function. Players are viewed as rational decision makers, strategies represent the actions available to each player, and the payoff function quantifies the rewards or losses that a player incurs following the execution of a specific action.

In the NB problem, the bargaining progress can be described as two or more players desiring more payoff through cooperation [30]. However, the payoff of each player is in conflict, so the bargaining progress may face the possibility of breaking down between different players. Based on this context, Nash proposed an NB solution, which is a unique solution that satisfies four specific axioms: Pareto optimality, symmetry, independence of irrelevant alternatives, and invariance to equivalent utility representations.

A simple NB problem typically involves a feasibility set X, a closed subset of $R^{2}$, which is usually assumed to be convex. The element x∈X is usually interpreted as the action a player can take. There is a payoff function u for some elements x∈X, denoted as u(x), $u\left(y\right)$,…, u(z), representing the payoff the player can receive after taking actions x, y,…, z. As mentioned earlier, a breakdown may occur in the bargaining progress. A point d represents the minimum payoff a player can accept, or in other words, point d is the payoff the player can obtain when a strategy of non-cooperation is adopted, and d is also called the disagreement point. In reality, there is at least one action x, such that u(x)≥d. If the bargaining game among n players can eventually reach an equilibrium point, Nash proved that the solution that satisfies four axioms is exactly the point ($x_{1}$, $x_{2}$, $x_{3}$,…, $x_{n}$) that maximizes the following expression:

(1) $$\max\left\{\left(u\left(x_{1}\right)-d_{1}\right)*\left(u\left(x_{2}\right)-d_{2}\right)*\left(u\left(x_{3}\right)-d_{3}\right)*\ldots *\left(u\left(x_{n}\right)-d_{n}\right)\right\}$$

The point ($x_{1}$, $x_{2}$, $x_{3}$,…, $x_{n}$) is called the NB solution, and it can be calculated as the point that maximizes the payoff of n players. 

### 2.3. Game Modeling

#### 2.3.1. Intersection Information

**Figure 1:**
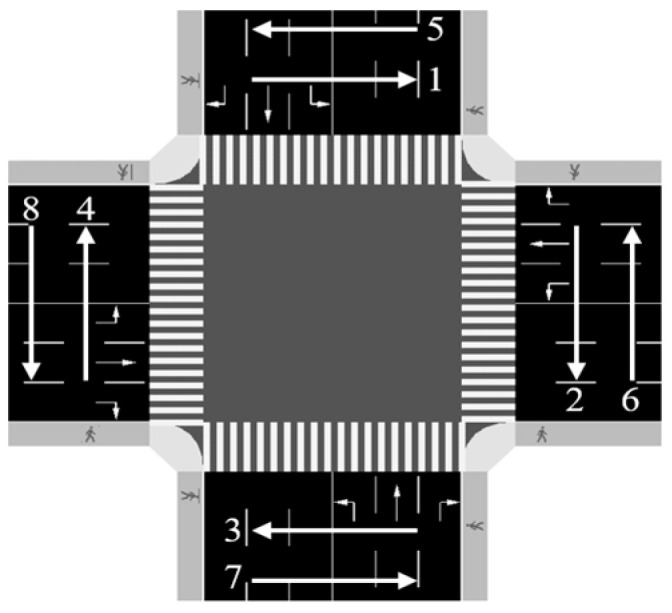
The tested intersection and pedestrian movements 1–8.

**Figure 2:**
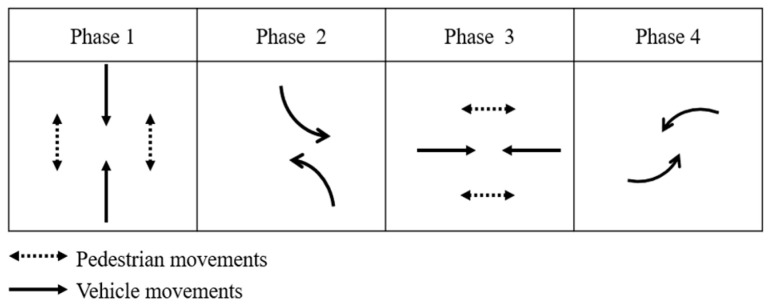
Phase sequence of the tested intersection.

The tested signalized intersection is modeled by SUMO software (1.18.0) with four approaches, and each approach comprises three lanes. Each lane is dedicated to either left-turning, through-moving, or right-turning vehicles. Pedestrians are assumed to have priority over turning vehicles at conflict points, so right-turning vehicles will stop and wait if their destination lane is blocked by moving pedestrians, which mimics real-world scenarios. The stopped right-turning vehicle will only start to accelerate once the pedestrians clear its lane. For pedestrian crossings, there are four crosswalks, each serving two opposing streams of pedestrians, so the pedestrians are categorized into eight movements based on their origin and destination (O-D) pairs. The layout of the tested intersection and pedestrian movements are shown in Figure 1. The phase sequence used in this intersection is shown in Figure 2. The detailed parameters of signal timing can be found in Table 1.

In a four-phase isolated intersection, each phase can be viewed as a player where conflicting payoff exists among them. However, they can also achieve greater payoff through cooperation. Thus, the signal control problem for an isolated signalized intersection can be modeled as an NB problem and then solved by an NB solution.

Let us define our game *G*:

(2) $$G=\left\{n,s,u\right\}$$

where n is the set of players, representing the four phases in an isolated signalized intersection, s is the strategies (actions) each player can select, and u is the payoff function. Since our goal is to determine the optimal green duration ($g_{i}$) between minimum green duration $\left(g_{min}\right)$ and maximum green duration $\left(g_{max}\right)$ for each phase, the strategies can be written as follows:

(3) $$s=\left\{g:g_{min}\leq g\leq g_{max}\right\}$$

#### 2.3.2. Payoff Function

The payoff function, a core component in the NB problem, was originally defined in a study [17] as the negative vehicle queue length of each phase. However, considering the inclusion of pedestrians in our scenario, we redefine the payoff function as the estimated negative weighted sum of “people” after taking a specific action. A conversion factor w is introduced, representing the average person occupancy per car. As referenced in a previous study [8], w is assigned the value of 1.54. The estimated weighted sum of people can be calculated according to the following equation:

(4) $$Q_{i}\left(g\right)=w*\left(\sum_{l\in i}{veh}_{t}^{l}+\sum_{l\in i}{veh}_{in}^{l}\right)+\sum_{j\in i}{ped}_{t}^{j}+\sum_{j\in i}{ped}_{in}^{j}-w*\sum_{l\in i}{veh}_{out}^{l}-\sum_{j\in i}{ped}_{out}^{j}$$

(5) $${∆t}_{i}=\sum_{1}^{i}g_{i}+\left(y+r\right)\left(i-1\right)$$

(6) $${veh}_{in}^{l}=\frac{{∆t}_{i}}{{∆t}_{veh}}*{veh}_{p}^{l}$$

(7) $${ped}_{in}^{j}=\frac{{∆t}_{i}}{{∆t}_{ped}}*{ped}_{p}^{j}$$

Specifically, the departure vehicles of lane l ${veh}_{out}^{l}$ is related to queued vehicles of lane l at time t ${veh}_{t}^{l}$ and green time g and arrival vehicles of lane l during ${∆t}_{i}$ ${veh}_{in}^{l}$. Let $g_{0}=({veh}_{t}^{l}+{veh}_{in}^{l})/{veh}_{dr}^{l}$, and we can give an estimated ${veh}_{out}^{l}$ by the following equation:

(8) $${veh}_{out}^{l}=\left\{\begin{array}{c} {veh}_{t}^{l}+{veh}_{in}^{l}+\frac{\left(g-g_{0}\right)}{{∆t}_{veh}}*{veh}_{p}^{l},\;\;g>g_{0}\\ {(veh}_{t}^{l}+{veh}_{in}^{l})*g/g_{0},\;\;g_{0}\leq g \end{array}\right.$$

The departure pedestrians of movement j ${ped}_{out}^{j}$ can be calculated similarly with ${veh}_{out}^{l}$. Let $g_{0}^{’}=({ped}_{t}^{j}+{ped}_{in}^{j})/{ped}_{j}^{l}$, and an estimated ${ped}_{out}^{j}$ can be given by the following equation:

(9) $${ped}_{out}^{j}=\left\{\begin{array}{c} {ped}_{t}^{j}+{ped}_{in}^{j}+\frac{\left(g-g_{0}^{’}\right)}{{∆t}_{veh}}*{ped}_{p}^{j},\;\;g>g_{0}^{’}\\ {(ped}_{t}^{j}+{ped}_{in}^{j})*g/g_{0}^{’},\;\;g_{0}^{’}\leq g \end{array}\right.$$

As mentioned above, the payoff function associated with selecting any other g∈s is as follows:

(10) $$u\left(g_{i}\right)=-Q\left(g_{i}\right)$$

#### 2.3.3. Disagreement Point

**Figure 3:**
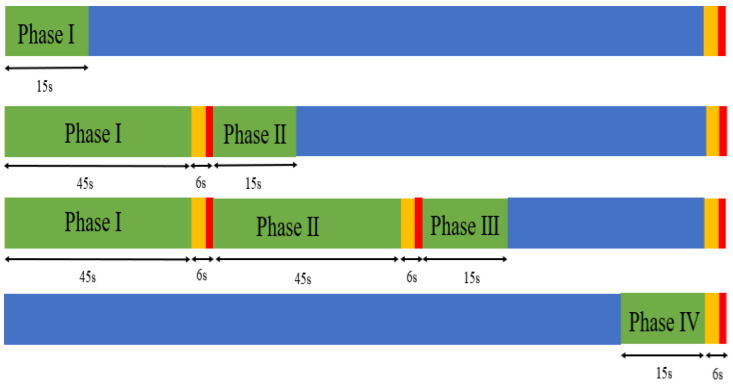
Calculation of the disagreement point.

The disagreement point refers to the minimum payoff that each player is willing to accept, and it significantly influences the ultimate solution. Each player evaluates the current and future circumstances to establish an acceptable minimum payoff. The strategy for each phase is to determine an optimal g between $g_{min}$ and $g_{max}$, considering that our signal control sequence is fixed. For Phase 1, in the first position, the minimum payoff is achieved by selecting $g_{min}$; hence, ${∆t}_{1}$ should be 15 s; for Phase 2, the minimum payoff can be defined as when Phase 1 selects $g_{max}$ and Phase 2 still only obtains $g_{min}$, so the ${∆t}_{2}$ is supposed to be 66 s; for Phase 3, both phases 1 and 2 select $g_{max}$, Phase 3 still only obtains $g_{min}$, and ${∆t}_{3}$ is 117 s; and Phase 4 is in the last position in one cycle, so the minimum payoff can be defined to choose the last 15 s of green time, so ${∆t}_{4}$ is 138 s. The calculation is shown in Figure 3.

(11) $$d_{1}=-Q_{1}\left(g_{min}\right)\;with\;{∆t}_{1}=15s$$

(12) $$d_{2}=-Q_{2}\left(g_{min}\right)\;with\;{∆t}_{2}=66s$$

(13) $$d_{3}=-Q_{3}\left(g_{min}\right)\;with\;{∆t}_{3}=117s$$

(14) $$d_{4}=-Q_{2}\left(g_{min}\right)\;with\;{∆t}_{4}=138s$$

Since each player is assumed to be rational, none of them desires the negotiation to collapse. Therefore, it is logical to select the minimum payoff among the four players as the disagreement point. In our model, we use $d_{min}=min(d_{1},d_{2},d_{3},d_{4})$ as the minimum payoff the players finally can accept, and then the disagreement point d can be defined as $d=\left(d_{min},d_{min},d_{min},d_{min}\right)$. After defining the payoff function and disagreement point, the objective is to minimize and equalize the weighted sum of “people” after taking actions across four phases. Thus, the NB solution $\left(g_{1}^{*},g_{2}^{*},g_{3}^{*},g_{4}^{*}\right)$ can be obtained through the optimization function below:

$$\max\prod_{1}^{4}\left(u\left(g_{i}\right)-d_{min}\right)$$

$$s.t.{\;g}_{min}\leq g_{i}\leq g_{max}$$

(15) $$\sum_{1}^{4}g_{i}=120$$

**Figure 4:**
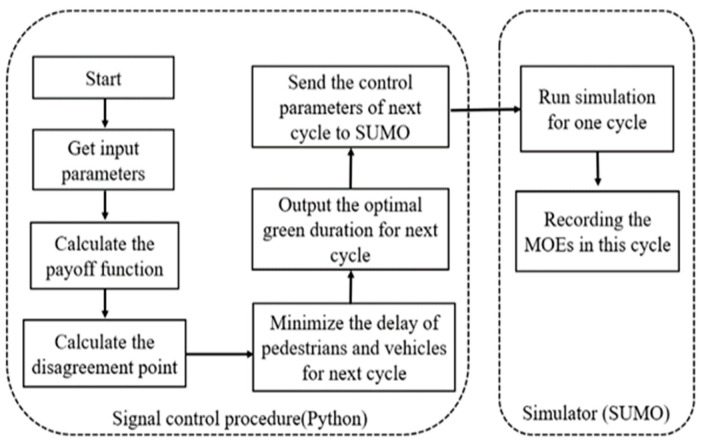
NB controller architecture.

After establishing the three essential elements required for GT, we incorporated the concept of NB into our model to address the signal control problem of an isolated signalized intersection. This approach takes both vehicles and pedestrians into account, with the objective of minimizing and equalizing the queued vehicles and pedestrians in each phase. Figure 4 shows the workflow of the NB approach process between Python and SUMO in a fixed cycle.

## 3. Results

### 3.1. Experiment Settings

**Figure 5:**
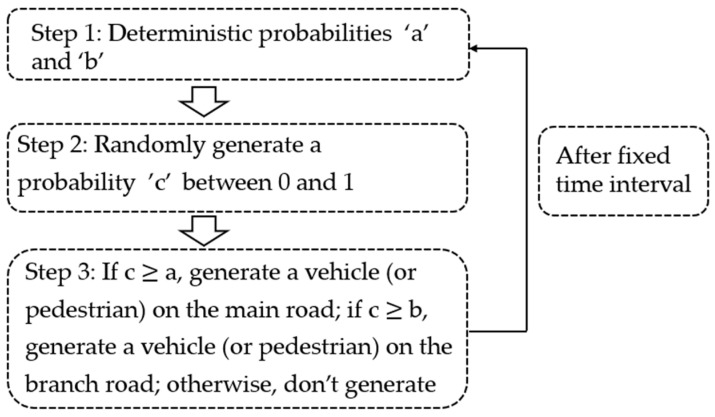
Vehicle and pedestrian generation workflow.

The simulation tests are carried out by the SUMO software, and pedestrians and vehicles are generated through the Traci interface in SUMO. About the pedestrian and vehicle generation settings, the workflow is shown in Figure 5. As Figure 5 shows, by varying the values of “a” and “b”, we can easily simulate different traffic demand scenarios. If the values of “a” and “b” are the same, a balanced traffic demand at the intersection is indicated. Conversely, different values for “a” and “b” can represent an unbalanced traffic demand at the intersection, such as the east–westbound representing the main road and the north–southbound representing the branch road. And since parameter “c” is randomly generated, it can help to reflect the stochastic traffic demand of the intersection to some extent. 

In SUMO, we can monitor the speed and position of vehicles and pedestrians in real-time, and when the speed of a vehicle and pedestrian at time t fall below a specific threshold, it is assumed that the vehicle is assigned to a queue and the pedestrian is waiting due to the red light. Then, the number of vehicles in the queue at time t of lane l (${veh}_{t}^{l}$) and the number of pedestrians waiting at time t for movement j (${ped}_{t}^{j}$) can be calculated. 

The simulation parameters are listed as follows:

1. Car-following behavior: IDM (Intelligent Driver Model);
2. Vehicle length: 4 m;
3. Vehicle maximum speed: 16.7 m/s;
4. Minimum gap between vehicles: 2 m;
5. Pedestrian walking speed: 1.4 m/s;
6. Vehicle threshold speed: 1.4 m/s;
7. Pedestrian threshold speed: 0.2 m/s;
8. Vehicle arrival time interval: 7.0 s;
9. Pedestrian arrival time interval: 9.5 s;
10. Traffic flow generation probability for the main road: a;
11. Traffic flow generation probability for a branch road: b.

In order to examine the performance of the proposed signal control algorithm, several measures of effectiveness (MOEs) are used as follows:

1. APD: Average pedestrian delay (s/ped), the average delay of each pedestrian due to a red light;
2. AVD: Average vehicle delay (s/veh), the average delay of each vehicle due to a red light;
3. AQL: Average queue length (veh), the average number of vehicles from the junction until the final vehicle in the queue;
4. ACE: Average CO_2_ emissions (g), the average amount of CO_2_ emitted by the vehicles;
5. AFC: Average fuel consumption (g), the average amount of fuel the vehicles use.

An actuated (Act) signal control strategy is used as a benchmark to evaluate the performance of the proposed NB signal control approach. Each simulation test runs for 1 h. Since “c” is randomly generated, to ensure robustness and reliability, each experimental scenario for each approach is simulated five times, and the mentioned MOEs of each time will be recorded. The final value of all MOEs is determined by calculating the average value of the five experimental results.

### 3.2. Results of Balanced Demand Scenarios

In this section, three comparative experiments are conducted to evaluate the performance of the proposed NB approach. the values of “a” and “b” are identical, and the values for the three experiments are 0.7, 0.75, and 0.8, respectively. The equality of “a” and “b” values signifies balanced traffic demand scenarios. The results are shown in Table 2.

As indicated in Table 2, the proposed NB signal control approach performs better than Act under different balanced demand scenarios. The NB approach achieves a maximum reduction of 17.93% in APD, with the reductions in APD consistently exceeding 12% across all three cases. The reductions in AVD range from 2.69% to 18.06%, indicating that the NB approach is able to reduce travel time for both pedestrians and vehicles, resulting in significant time benefits. Furthermore, the reductions in AQL range from 7.18% to 20.83%, and the reductions in ACE and AFC range from 1.25% to 8.92%. The results for fuel consumption and CO_2_ emissions also reveal that the NB approach is more cost-effective and environmentally friendly. In different balanced demand scenarios, all of the proposed NB approaches exhibit better performances than the Act approach. 

It can be noted that when probabilities are 0.7, the reductions of AVD, AQL, and AFC are slight, and this is mainly attributed to the fact that unlike the Act control strategy, which only considers vehicles, the NB approach also takes pedestrians into account. However, as traffic demand increases, the NB approach shows a more pronounced reduction in average queue length.

**Figure 6:**
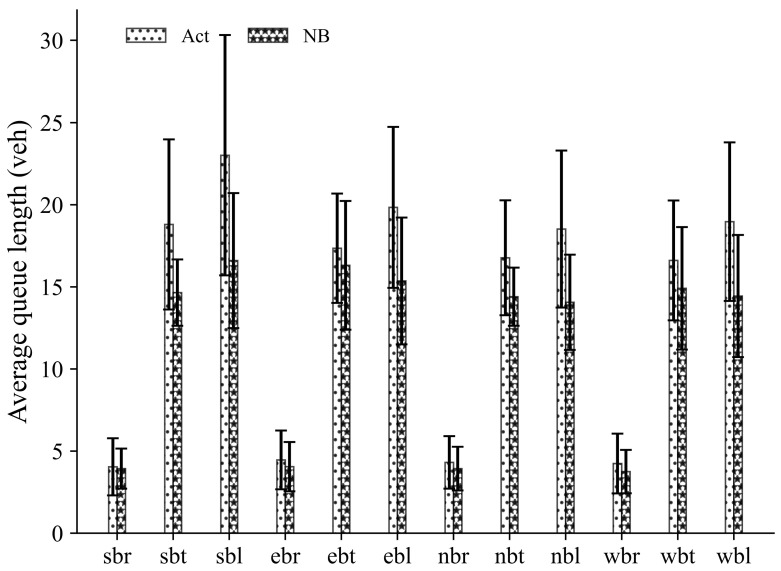
Average queue length for all movements under a balanced demand scenario. Note: nbt/wbt = northbound/westbound (through moving); nbl/wbl = northbound/westbound (left turning); nbr/wbr = northbound/westbound (right turning).

In order to provide more detailed information on AQL under a balanced demand scenario, we give a more detailed analysis (when the probabilities are 0.75). The average value and standard deviation across all movements over the entire simulation time for different control strategies are shown in Figure 6. The results demonstrate that compared with the Act approach, the NB approach demonstrates a significant reduction in both average values and standard deviation of the vehicle queue length for all movements, proving evidence for a greater stability of the proposed NB control strategy. 

### 3.3. Results of Unbalanced Demand Scenarios

**Figure 7:**
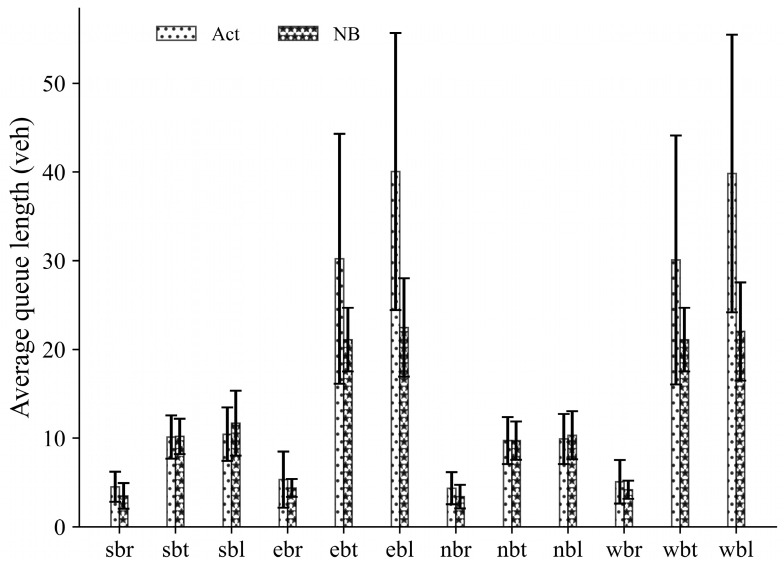
Average queue length for all movements under the unbalanced demand scenario. Note: nbt/wbt = northbound/westbound (through moving); nbl/wbl = northbound/westbound (left turning); nbr/wbr = northbound/westbound (right turning).

In this section, we conduct several experiments to evaluate the performance of the proposed NB signal control approach under unbalanced demand scenarios. The arrival time interval of vehicles and pedestrians remains constant at 7.0 s and 9.5 s. In the unbalanced demand scenarios, we consider eastbound and westbound as the main road and northbound and southbound as the branch road. The vehicle and pedestrian arrival probability of the main road is set as 0.8, 0.9, and 1.0, while on the branch road, the arrival probability is set as 0.7, 0.6, and 0.5, respectively. The results are presented in Table 3. Detailed average values and standard deviations across all movements when a = 1.0 and b = 0.5 are shown in Figure 7.

As illustrated in Table 3, when the unbalanced degree increases, the reductions in APD decrease, and when the vehicle and pedestrian flow on the main road are approximately twice that of the branch road, the reduction in APD is 3.79%; this is an acceptable outcome. The reductions in AVD and AQL are significant, with the maximum reduction reaching 31.15% and 32.28%, respectively. From economic and environmental perspectives, the reductions in ACE and AFC range from 11.80% to 16.22%. These results also demonstrate the superiority of the proposed NB control approach under different unbalanced demand scenarios. Furthermore, the observed pattern aligns with that seen in the balanced demand scenarios, indicating that the greater the degree of unbalance, the more pronounced the reduction in APD. 

Notably, an interesting phenomenon can be observed in Figure 7, where the NB approach demonstrates a significant reduction in both average values and standard deviation of queue length on the main road, with the maximum reduction occurring in westbound left turns, reaching 44.69%. However, the results in some lanes of the branch road are opposite to those on the main road; the southbound and northbound left-turning queue lengths of the NB approach are higher than the Act control strategy. This interesting phenomenon is also found in a previous study [25]. Firstly, this might be attributed to random arrivals for the northbound and southbound movements. Secondly, in our NB approach, each phase is modeled as a rational player aiming to obtain the maximum payoff through cooperation; therefore, when the degree of unbalanced demand is relatively high, the phases on the branch road may make certain sacrifices for the overall payoff, illustrating a manifestation of cooperation.

Moreover, when applying the NB control strategy, the average queue length on each lane exhibits minimal variation, and the maximum and minimum average queue lengths on the main road are 22.47 and 21.10, respectively. However, under the actuated control strategy, the maximum and minimum average queue lengths on the main road are 40.04 and 30.08, respectively. These numbers also illustrate that the NB approach fully considers the payoff of each phase, ultimately achieving a more balanced queue length for each phase compared to the actuated control strategy.

### 3.4. Sensitivity Analysis of Right-Turning Vehicles

In most signal control strategies, dedicated lanes for right-turning vehicles often lead to their exclusion from detailed consideration. However, in our scenario, pedestrians are also taken into account, leading to potential conflicts between pedestrians and right-turning vehicles. It is evident that allocating excessive time to pedestrian crossing phases (Phase 1 and Phase 3 in our scenario) can increase the likelihood of conflicts, potentially causing significant delays for right-turning vehicles. Thus, this section conducts a straightforward sensitivity analysis to evaluate the performance of the NB signal control approach under the balanced demand scenario mentioned above. Both parameters “a” and “b” are set to 0.8, and we continue to maintain the pedestrian arrival time interval of 9.5 s, and the arrival time interval of left-turning and through-moving vehicles is 7.0 s. We are examining various arrival time intervals for right-turning vehicles, including the intervals of 3.6 s, 4.8 s, 7.2 s, and 14.4 s, respectively. Each simulation runs for 1 h, and the results, including the MOEs of right-turning vehicles and the average delay of pedestrians, are recorded and presented in Table 4. 

As indicated in Table 4, the NB approach exhibits a significant decrease in APD of approximately 18% compared to the Act control. Moreover, the delay and queue length of right-turning vehicles decreased by more than 20%, with notable improvements in the reduction in CO_2_ emissions and fuel consumption. Significantly, when the volume of right-turning vehicles exceeds a specific threshold, the proposed NB approach demonstrates a substantial reduction in delay and queue length for right-turning vehicles. 

Specifically, the reduction in delay and queue length is 37.16% and 34.50%, respectively, while the decrease in CO_2_ emissions and fuel consumption reaches 17.85%. Due to the NB approach models, each phase is a rational player, and careful consideration is given to the payoff of each player, including Phase 2 and Phase 4, which serve left-turning vehicles only. Intuitively, the payoff of right-turning and left-turning vehicles are aligned since they do not conflict, safeguarding the payoff of Phase 2, and Phase 4 indirectly protects the payoff of right-turning vehicles as well. Although there is also no conflict between right-turning and through-moving vehicles, allowing the through-moving phases (Phase 1 and Phase 3), which also permit pedestrian crossing, to excessively prioritize the payoff of through-moving vehicles increases the likelihood of conflicts between right-turning vehicles and pedestrians, and this leads to an increase in delay for right-turning vehicles. Consequently, the NB approach consistently delivers favorable outcomes, even in scenarios characterized by high demand for right-turning movements.

## 4. Conclusions and Future Work

This paper develops an NB-based signal control approach for isolated intersections, taking into account both vehicles and pedestrians in a connected environment. We initially model four phases in the intersection as four rational players, incorporating both vehicles and pedestrians into our payoff function. Subsequently, the minimum payoff for each player is defined, with the objective of minimizing and equalizing queued pedestrians and vehicles across different phases, and the optimal green duration for each phase during a fixed cycle length is then determined through the application of the NB solution.

To assess the effectiveness of the proposed NB approach, an actuated signal control strategy is chosen as a benchmark. Several simulation experiments are conducted by SUMO software under various traffic demand levels and demand patterns. By introducing the random parameter “c”, we have taken into account the influence of stochastic traffic demand. The simulation results demonstrate that the NB approach outperforms the benchmark in terms of average delay, queue length, CO_2_ emissions, fuel consumption for vehicles, and the average delay of pedestrians. Additionally, detailed analyses are conducted in balanced and unbalanced demand scenarios to affirm the superiority of the proposed NB control approach, and a detailed examination of vehicle queue length in each lane is performed to showcase the enhanced stability of the NB approach. The proposed NB approach consistently achieves more equalized queue length for each lane compared to the actuated control strategy, indicating a higher degree of fairness for each participant. Furthermore, a sensitivity analysis is carried out for right-turning vehicles, with simulation results indicating superior performance, particularly when the demand for right-turning vehicles exceeds a certain threshold.

We must acknowledge that the consideration of stochastic traffic demand in this paper is insufficient. The method we employ may not fully capture the highly stochastic traffic demand in the real world. Future research could explore this aspect further. Additionally, utilizing data in realistic scenarios to better reflect actual traffic conditions is also crucial.

## Figures and Tables

**Table 1:** Notation.

Symbol	Definition
c	a cycle length of 144 s
$g_{min}$	a minimum green duration of 15 s
$g_{max}$	a maximum green duration of 45 s
$g_{i}$	green duration for phase i
y	a yellow duration of 4 s
r	a red duration of 2 s
w	a conversion factor of 1.54
i	phase index of the intersection
t	the beginning time of each cycle
${∆t}_{veh}$	vehicle arrival time interval
${∆t}_{ped}$	pedestrian arrival time interval
${veh}_{p}^{l}$	vehicle arrival probability of lane l
${ped}_{p}^{j}$	pedestrian arrival probability of movement j
${∆t}_{i}$	time interval between t and the end of the green time of phase i
${veh}_{t}^{l}$	the number of queued vehicles of lane l at time t
${veh}_{in}^{l}$	the number of arrival vehicles of lane l during ${∆t}_{i}$
${veh}_{out}^{l}$	the number of departure vehicles of lane l
${veh}_{dr}^{l}$	the vehicle departure rate of lane l
${ped}_{t}^{j}$	the number of queued pedestrians of movement j at time t
${ped}_{in}^{j}$	the number of arrival pedestrians of movement j during ${∆t}_{i}$
${ped}_{out}^{j}$	the number of departure pedestrians of movement j
${ped}_{dr}^{j}$	the pedestrian departure rate of movement j
$Q_{i}\left(g\right)$	estimated weighted sum of people after applying a green time g for phase i

**Table 2:** Simulation results of balanced demand scenarios.

Probabilities		a = 0.7 b = 0.7	a = 0.75 b = 0.75	a = 0.8 b = 0.8
APD	Act	59.10	63.98	64.84
	NB	51.73	53.20	53.21
Reduction		12.47%	16.85%	17.94%
AVD	Act	50.93	66.61	102.29
	NB	49.56	54.58	86.34
Reduction		2.69%	18.06%	15.59%
AQL	Act	10.86	13.91	20.55
	NB	10.08	11.37	16.27
Reduction		7.18%	18.26%	20.83%
ACE	Act	289.24	328.10	411.66
	NB	285.63	299.10	374.92
Reduction		1.25%	8.84%	8.92%
AFC	Act	92.26	104.25	131.31
	NB	91.11	95.04	119.60
Reduction		1.25%	8.84%	8.92%

**Table 3:** Simulation results of the unbalanced demand scenario.

Probabilities		a = 0.8 b = 0.7	a = 0.9 b = 0.6	a = 1.0 b = 0.5
APD	Act	62.72	59.23	53.30
	NB	52.88	52.73	51.28
Reduction		15.69%	10.97%	3.79%
AVD	Act	69.36	81.34	85.39
	NB	53.29	56.00	60.50
Reduction		23.17%	31.15%	29.15%
AQL	Act	14.37	16.24	17.72
	NB	11.18	11.52	12.00
Reduction		22.20%	29.06%	32.28%
ACE	Act	334.21	359.44	366.47
	NB	294.76	301.25	311.80
Reduction		11.80%	16.19%	14.92%
AFC	Act	106.60	114.70	116.89
	NB	94.02	96.09	99.45
Reduction		11.80%	16.22%	14.92%

**Table 4:** Simulation results of the right-turning vehicle sensitivity analysis.

Time Interval		3.6 s	4.8 s	7.2 s	14.4 s
APD	Act	65.10	64.95	64.94	64.68
	NB	53.63	53.28	53.22	52.98
Reduction		17.62%	17.97%	18.05%	18.09%
AVD	Act	72.50	21.10	16.43	12.86
	NB	45.56	16.46	12.74	10.17
Reduction		37.16%	21.99%	22.46%	20.92%
AQL	Act	26.26	8.63	5.09	2.41
	NB	17.20	6.86	4.04	1.78
Reduction		34.50%	20.51%	20.63%	26.20%
ACE	Act	309.17	209.24	200.66	194.09
	NB	253.99	198.97	192.16	187.49
Reduction		17.85%	4.91%	4.24%	3.40%
AFC	Act	98.61	66.74	64.00	61.91
	NB	81.01	63.46	61.29	59.80
Reduction		17.85%	4.91%	4.23%	3.41%

## Data Availability

Data are contained within the article.
